# Multimorbidity: What do we know? What should we do?

**DOI:** 10.15256/joc.2016.6.72

**Published:** 2016-02-17

**Authors:** Rokas Navickas, Vesna-Kerstin Petric, Andrea B. Feigl, Martin Seychell

**Affiliations:** ^1^Vilnius University, Faculty of Medicine, Vilnius, Lithuania; ^2^Vilnius University Hospital Santariškiu˛ Klinikos, Vilnius, Lithuania; ^3^Ministry of Health, Republic of Slovenia, Ljubljana, Slovenia; ^4^Harvard T.H. Chan School of Public Health, Boston, MA, USA; ^5^Abt Associates, Cambridge, MA, USA; ^6^Directorate General for Health and Food Safety, European Commission, Brussels, Belgium

**Keywords:** Multimorbidity, multiple chronic conditions, comorbidity, European Union, European Commission, JA-CHRODIS, Slovenia, ICARE4EU

## Abstract

Multimorbidity, which is defined as the co-occurrence of two or more chronic conditions, has moved onto the priority agenda for many health policymakers and healthcare providers. Patients with multimorbidity are high utilizers of healthcare resources and are some of the most costly and difficult-to-treat patients in Europe. Preventing and improving the way multimorbidity is managed is now a key priority for many countries, and work is at last underway to develop more sustainable models of care. Unfortunately, this effort is being hampered by a lack of basic knowledge about the aetiology, epidemiology, and risk factors for multimorbidity, and the efficacy and cost-effectiveness of different interventions. The European Commission recognizes the need for reform in this area and has committed to raising awareness of multimorbidity, encouraging innovation, optimizing the use of existing resources, and coordinating the efforts of different stakeholders across the European Union. Many countries have now incorporated multimorbidity into their own healthcare strategies and are working to strengthen their prevention efforts and develop more integrated models of care. Although there is some evidence that integrated care for people with multimorbidity can create efficiency gains and improve health outcomes, the evidence is limited, and may only be applicable to high-income countries with relatively strong and well-resourced health systems. In low- to middle-income countries, which are facing the double burden of infectious and chronic diseases, integration of care will require capacity building, better quality services, and a stronger evidence base.

## Introduction

The number of people affected by multiple chronic diseases (multimorbidity) is increasing dramatically around the world, and caring for them has placed considerable strain on many health systems. People with multimorbidity have complex care needs and are some of the most costly and challenging patients to manage. Although it has at last been recognized that more sustainable models of care for multimorbidity should be introduced as a matter of urgency, policymakers and healthcare providers need good quality evidence on which to build the case for change. But how much do we really know about multimorbidity? Do we know enough to help us prioritize patients, intervene most appropriately, and apply the best and most cost-effective models of care? In this article, this important question is addressed from four different perspectives: the clinician, the European Commission, the health policymaker and the health economist.

## Understanding the epidemiology, risk factors, and consequences of multimorbidity

We have been working hard to improve the management of chronic diseases for many decades, with major investments made across the board. Today, however, as budgets tighten and every expenditure must be scrutinized and defended, the focus is turning towards those patients generating the highest costs, but experiencing the least benefit from healthcare systems. In a nutshell, these are the patients with multiple chronic conditions – those with “multimorbidity”.

**Figure 1:**
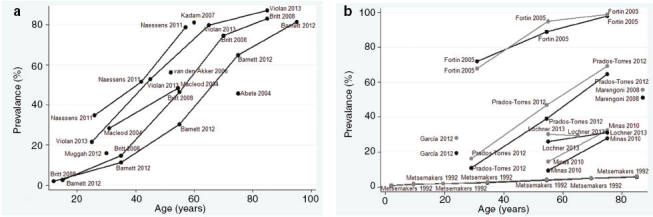
Prevalence of multimorbidity by age group: overall (a) and by sex (b) in primary care studies identified in a systematic review of the literature. Reproduced from Violan C *et al*. Prevalence, determinants and patterns of multimorbidity in primary care: a systematic review of observational studies. PLoS One 2014;9(7):e102149 [3].

Multimorbidity is defined as the co-occurrence of two or more chronic conditions [1, 2] and has been estimated to affect up to 95% of the primary care population aged 65 years and older [3]. Although the prevalence of multimorbidity increases with age, it is not exclusively a condition affecting the elderly, with many studies reporting high rates of multimorbidity amongst working-age populations (Figure 1) [3].

Risk factors for multimorbidity have not been well studied. Ageing is the most consistent and potent risk factor, and it has recently been proposed that multimorbidity may be the result of a multisystem loss of reserve and function that leads to a low-grade proinflammatory state, multiple hormonal dysregulation, and an increased susceptibility to chronic diseases [4, 5]. Women and those with a lower socioeconomic status appear especially prone to developing multimorbidity [3], although the reasons for this are not yet clear. A recent 10-year follow-up study conducted in Finland reported that predisposing factors for multimorbidity amongst a disease-free population were smoking, physical inactivity and high body mass index, with hypertension and low level of education as additional risk factors reported in men [6]. Other studies have found a clear association between obesity and multimorbidity [7–10], with one study demonstrating that accumulating unhealthy lifestyle factors progressively increases the risk of multimorbidity [9].

The consequences of multimorbidity are wide-ranging and severe. People with multimorbidity die prematurely [11]; they have more frequent hospital admissions and longer hospital stays [12]; and they see a large number of different medical specialists during a typical year [12]. A recent analysis of the direct costs of multimorbidity in the United States Veterans Affairs Health Care System reported that, of the 5% of the highest-cost patients in the system (who accounted for 47% of total healthcare costs), approximately two-thirds had multiple chronic conditions [13].

Multimorbidity also profoundly affects an individual’s well being, quality of life, and ability to function normally [14]. In studies conducted up to 2003, an inverse relationship between multimorbidity and overall quality of life was reported; however, the closest association observed was the effect of multimorbidity on physical functioning [14]. Reduced physical functioning may lead to the development of depression and other affective disorders, adding to the medication burden associated with multimorbidity. Patients with multimorbidity struggle to managing the multiple medications prescribed to them [15], leading to difficulties with treatment adherence, and further reduction in quality of life.

Despite the increasing numbers of patients with multimorbidity, clinical practice guidelines and delivery of care are still primarily built around single diseases, which can have many undesirable effects [16, 17]. To deliver better and more cost-effective care for our patients with multimorbidity, we must shift the paradigm from vertical monomorbid approaches to horizontal multimorbid ones. Our services need to be reorganized to deliver individualized and more structured care, better care co-ordination and management, enhanced multidisciplinary teamwork, and greater support for patient education and self-management – optimizing the use of 21st century technology solutions wherever we can. Clinical practice guidelines will need to be developed that are relevant to the needs of patients with multimorbidity, and which will require complex treatment decisions to be made in the absence of high-quality or direct evidence [17].

During this time of considerable change, we will have to accept that extra investment will initially be required while our focus moves onto primary prevention, the management of younger patients with multimorbidity, and the development of healthcare systems that are better equipped to meet the needs of older patients. It is believed that, within 20 or so years, this investment will reap rewards, bringing tangible benefits to individuals, populations, and healthcare systems.

## Improving our understanding of multimorbidity: what is the European Commission doing?

It has been estimated that at least 50 million people in the European Union (EU) have multimorbidity, and this number is expected to increase further as the population ages [18]. Multimorbidity reduces life expectancy, affects the individual’s quality of life and ability to work, increases the risk of hospitalization, and leads to excessive use of other healthcare resources [19]. Ultimately, multimorbidity impacts the sustainability of health and social care systems.

Health systems have traditionally been disease-oriented, focusing on curing or managing individual acute and chronic conditions. Although most patients with multimorbidity are affected by common conditions – such as hypertension, ischaemic heart disease, and diabetes, none of which are rare and all of which are individually treatable – the problem originates from the failure to adequately accommodate the interplay between them. Indeed, the disease-oriented approach does not work for the patient with multimorbidity, resulting in fragmented, inefficient, and ineffective care. If we are to improve the resilience and sustainability of health systems, a paradigm shift will be required towards a more patient-centred model that addresses the needs of theses individuals in a more holistic way, focusing on multidisciplinary, integrated, and coordinated care. Achieving this will require innovation and reform at all levels of the political and healthcare systems.

The European Commission is ideally placed to contribute to these reforms by raising awareness of multimorbidity, encouraging innovation, optimizing the use of existing resources, and bringing together and coordinating the efforts and expertise of different stakeholders. Multimorbidity has been identified as a key issue by the European Innovation Partnership on Active and Healthy Ageing, which was established in 2011 to improve the health of our ageing population [20]. The Partnership’s three priority areas for action are: (1) prevention, screening, and early diagnosis; (2) care and cure; and (3) active ageing and independent living. Action Groups have been established to research, compile, and disseminate good practices throughout Europe in several areas, including prescription and adherence to medical plans and integrated care – both of which address important aspects of the management of multimorbidity. In its first review of good practices across Europe [21], the Action Group on Prescription and Adherence to Medical Plans identified a major issue with polypharmacy and poor medication adherence amongst patients with multimorbidity, highlighting the increased risk of adverse events and hospitalization associated with inappropriate prescribing. Preliminary work has also been completed to identify successful interventions, such as medication review and reconciliation aimed at reducing polypharmacy, and to identify inappropriate prescriptions in patients with multimorbidity, and these are summarized in the Group’s 2013 report [21]. In addition, as a result of the collaborative work dynamics established in the Action Group, a follow-up collaboration has been established through the Stimulating Improvement Management of Polypharmacy and Adherence in The Elderly (SIMPATHY) project, an EU-funded activity aiming to stimulate, promote, and support innovation across the EU in the management of appropriate polypharmacy and adherence in the elderly, in order to contribute to efficient and sustainable healthcare systems [22]. The Action Group on Integrated Care has focused on practical tools to support local service delivery, mapping innovative solutions for the management of chronic diseases to improve the quality and sustainability of services [23].

The European Commission has also funded the Innovating Care for People with Multiple Chronic Conditions in Europe (ICARE4EU) project [24], under the framework of its Health Programme 2008–2013, to provide insight into current practices of integrated care for people with multimorbidity in European countries [18] (see also Albreht *et al.* [25]). The project has so far analysed integrated care activities in 31 European countries, with 101 innovative approaches identified to date [26].

More recently, the European Commission has initiated the Joint Action on Chronic Diseases and Promoting Healthy Ageing Across the Life Cycle (JA-CHRODIS) [27], bringing together over 60 collaborating partners from 26 member states. These partners are working together to identify, validate, exchange, and disseminate good practice across the EU, with a focus on health promotion and primary prevention of chronic disease. In recognition of the failings of current models to adequately address multimorbidity, a work package on multimorbidity has been established within the JA-CHRODIS, with the aim of advising on the best possible patient-centred care model for patients with multimorbidity, taking into account outcomes, cost-effectiveness, applicability, and replicability.

Considering multimorbidity in a broader context, one of the Commission’s highest current priorities is the issue of health system performance assessment. In September 2014, the Council Working Party on Public Health at Senior Level adopted the Terms of Reference from an Expert Group on Health Systems Performance Assessment (HSPA), which provides member states with a forum for exchange of experiences on the use of HSPA at a national level. A subgroup on integrated care has been established that will contribute to designing a framework for performance assessment of integrated care, paving the way to supporting national policymakers by identifying tools and methodologies for developing HSPAs. This will eventually serve as a basis on which to determine whether the integrated care interventions we are designing are performing well for patients with multimorbidity.

The findings from many of these initiatives were discussed at a recent European Commission conference entitled, “*Which priorities for a European policy on multimorbidity?*”, held in Brussels on October 27, 2015 [28]. The aim of the conference was to share experiences and practices in the management of multimorbidity, to learn from innovative healthcare approaches, and to explore how we can overcome the barriers to a common framework in multimorbidity. The conference represented a milestone in the development of EU public policies on multimorbidity, creating a common engagement across stakeholders aimed at addressing multimorbidity at a European level. The stakeholders at the meeting identified priorities in order to move towards building a common framework on multimorbidity. These priorities are summarized in Table 1 [28].

The European Commission is committed to addressing all aspects of multimorbidity and to developing policies and guidance that are evidence-based and relevant across the EU and within its member states. National policymakers are encouraged to use the work of the European Commission to help inform their own policies and to contribute to the cross-fertilization of ideas and exchange of best practices in this field.

## Making a national commitment to preventing multimorbidity: the Slovenian experience

Slovenia is a central European country bordering Italy, Austria, Croatia, and Hungary, with a population of just over 2 million. The country was formally a constituent part of Yugoslavia, but declared independence in June 1991, joining the EU in May 2004. Slovenia has a democratic political system with a parliamentary form of state power. The country has enjoyed continuous economic growth since 1992, and is now ranked among the most developed countries in the world in terms of its economy.

The health system of Slovenia has undergone a major transformation since the country achieved independence. Fundamental reforms aimed at modernizing the health system were implemented in 1992, with the introduction of compulsory health insurance, an approval process for the provision of private healthcare, the introduction of co-payments for healthcare services, and the reintroduction of professional associations [29]. Today, as a result of these and many other subsequent reforms, Slovenia has a modern, health insurance-based system that is built around country-wide primary care providers that aim to offer integrated healthcare delivered within the local community [29].

### A rapidly ageing population

As in many other central and eastern European countries, the population of Slovenia is ageing rapidly. The birth rate has decreased from 15.7 per 1,000 population in 1980 to 10.3 per 1,000 population in 2014, while at the same time, life expectancy has increased to just below the EU average of 80.6 years (in 2013) [30]. As a result, since the early 1990s, the elderly population (aged 65 years and older) has increased by approximately 60%, and by 2014, elderly people accounted for 17.2% of the total population [31]. Unsurprisingly, the prevalence of multimorbidity in Slovenia is also increasing [32].

### Addressing multimorbidity through prevention and coordinated care

The rapidly ageing population poses a major challenge to the health system of Slovenia, and significant work is underway to meet the increasing demands placed on the system by the rising incidence of chronic conditions and multimorbidity, and by the increasing rates of obesity and other risk factors within our population.

The most recent national healthcare plan, highlighted the prevention and management of chronic diseases as key areas for strategic development, with health enhancement programmes recommended to tackle common risk factors, such as the harmful use of alcohol, tobacco, unhealthy diets, and physical inactivity, and poor mental health [33]. A new proposal of the health plan has recently been adopted by the Government and is now pending adoption by the Parliament. In this proposal, the ageing population and related multimorbidity and non-communicable diseases (NCDs) are recognized as the main challenges for the sustainability of the health system. Some of the priority areas of action in optimizing healthcare include the integration of services; upgrading primary healthcare and mental health services; empowering patients; investing in preventive services; and all of the society approach to risk factors.

An analysis of the system, in cooperation with the European Observatory for Health Systems and Policies and the World Health Organization, has now been completed. The analysis has shown that Slovenia has a robust primary healthcare that offers a good foundation to address the changing health and healthcare needs of the population; but it faces the challenge of fragmentation of service organization and delivery.

In addition, when looking for appropriate solutions to better control NCDs, we have learned a lot from the care of patients with diabetes, a common and potentially preventable chronic condition that requires demanding and complex care. By bringing together experts from different specialties and patients – and learning from both – we have been able to develop a National Diabetes Strategy by 2020 that puts patients firmly at the centre, and builds on the principles of empowering patients, fostering partnership, coordinating care across different providers, both vertically and horizontally, and ensuring ongoing follow-up through a coordinating group and action plans [34]. In developing the plan, our goals were to reduce the prevalence of diabetes, ensure its early detection, and reduce the incidence and impact of complications – goals that could readily be applied to the management of most long-term conditions.

To tackle multimorbidity, in particular, we have focussed our planning on several key areas. Firstly, we recognized that both social deprivation and mental health problems increase the risk of multimorbidity [35], so we are committed to ensuring that social and healthcare providers work more closely to address these issues more effectively. Patients with mental health problems receive treatment from a broad range of professionals, including psychiatrists and home nurses, who serve to connect the patient with hospital, community, and social care providers, and to monitor the patient’s condition and medications [36]. In addition to receiving both acute and long-term treatments, patients with mental health conditions engage in group therapies and disease-prevention programmes, receive occupational therapy, and participate in programmes that promote independence and social inclusion and reintegration.

Secondly, since the prevalence of multimorbidity continues to rise across our population and within younger age groups, we are investing further to strengthen our prevention programmes. Health promotion and education have been at the heart of our endeavours to reduce the burden of NCDs and multimorbidity since 2002, and The National Public Health Institute, with its Centre for the Management of Prevention Programmes and for Health Promotion designs, prepares, and monitors national prevention and screening programmes, including those targeted towards lifestyle interventions. Health promotion and education programmes are also delivered at the primary care level by nurses and other healthcare professionals in health education centres within the community health centres.

In 2011, we launched the concept of “model practices” aimed at enhancing the role of primary care practices in the prevention of chronic conditions and encouraging lifestyle changes. These practices are supported by registered nurses who work part-time in family medicine practices to educate patients with chronic diseases/multimorbidity on risk-factor management and self-care. Our goal is to increase the time available for these activities within each model practice and to expand the service across all primary care providers. Model practices play a key role in navigating patients between specialists, thereby reducing duplication and omission, and improving service efficiency.

Finally, we are addressing the needs of vulnerable population groups with multiple risk factors and chronic conditions who do not attend services offered in primary healthcare. There is evidence that multimorbidity is more prevalent in people from lower socioeconomic groups [35]. By strengthening community nursing in primary care and introducing protocols for cooperation between primary and secondary care teams and with social services, we aim for better integration of services, including social care services, in this regard.

We have witnessed unparalleled change in the Slovenian healthcare system over the past 25 years, and the system will continue to evolve as new challenges emerge and we refocus our efforts where they are needed most. There are many hurdles to overcome before we are delivering effective and cost-effective care to our patients with multiple chronic conditions; however, we are committed to achieving this and are moving in the right direction.

## How cost-effective is integrated care for chronic conditions? How much do we really know?

The recent announcement by the United Nations of their sustainable development goals for the 2030 Agenda for Sustainable Development has propelled universal healthcare and NCDs into the international development spotlight [37]. This has the potential to shift the health development agenda away from one that is disease-specific, with vertical funding and approaches to implementation, to more integrated, systems-based approaches to achieve “health for all”. This is a welcome development. However, while integration of services seems logical for high-capacity health systems, as a global community, we know very little about the cost-effectiveness of integrated care approaches, and even less about the cost-effectiveness of treating multimorbidity, or the benefits of integrated care for these patients. Emerging evidence from a rare, randomized controlled study conducted in the United States, revealed that transitioning from a disease-focused model to one of collaborative care delivery was indeed cost-effective, and helped to eliminate cost redundancies when managing patients with multiple chronic conditions [38]. This primary-care-based study used a randomized design to evaluate the ability of a systematic care management programme aimed at improving depression, haemoglobin A_1C_ , systolic blood pressure, and low-density lipoprotein cholesterol in patients with poorly controlled diabetes mellitus, coronary heart disease (or both), and comorbid depression. Patients were randomized to receive usual care or an enhanced collaborative care intervention in which nurse care managers worked with patients and primary care physicians to provide treatment of multiple-disease risk factors [38]. By 24 months post-randomization, compared with the control group, the intervention patients had experienced an average of 114 additional depression-free days, and an estimated 0.335 additional quality-adjusted life-years. Average outpatient cost savings of US $594 per patient over 2 years were reported. This study provides encouraging evidence that integrated care for people with multimorbidity may be cost-effective, and the results of other ongoing studies (e.g. the MPI_AGE European Project [39]) may shed further light on this issue.

While high-income countries are working to redefine how existing health systems can address multimorbidity most efficiently, low- and middle-income countries are facing additional challenges. For these countries, the battle to combat infectious diseases is ongoing, against a backdrop of weak health systems and vertically focused donor approaches. Unlike in high-income countries, the burden of multimorbidity in countries with low or middle incomes includes the immediate and long-term consequences of infectious diseases. In South Africa, for example, the prevalence of human immunodeficiency virus in adults is close to 30%, leading to widespread premature ageing and the increased risk of cardiovascular disease associated with some antiretroviral treatments. Overweight and obesity are prevalent and largely uncontrolled in both the general population and amongst those infected with human immunodeficiency virus, and hypertension rates are also rising. Training for community healthcare workers to recognize and treat the symptoms of, and risk factors for, chronic conditions is suboptimal, and most healthcare professionals are already working at full capacity. A similar picture emerges from other regions of Africa and other low-income countries.

In situations such as these, where poverty and a low capacity coexist, questions remain over the value of integrating infectious and chronic disease care. A systematic review of the impact of integrating primary healthcare services in low- and middle-income countries found that, while there was some evidence that adding extra services or creating links between existing services improved the delivery of healthcare, there was no evidence that better integration improved health status [40]. Indeed, in some cases, integration led to deterioration in service delivery.

For well-financed, high-quality health systems that have the capacity, knowledge, and ability to share that knowledge, it seems likely that integrating care for people with multimorbidity will create efficiency gains and improve health outcomes. In contrast, for developing countries facing the double burden of infectious and chronic diseases, integration of services will need to be matched by capacity assessments and capacity building, better quality services, and a stronger body of evidence. Unfortunately, it seems the world may not yet be ready for a global approach to improving the management of multimorbidity, and the goal of sustainable health systems and health for all, as set forth by the sustainable development goals, are a long way off.

## Summary and conclusions

Considering the major impact of multimorbidity on individuals and healthcare systems, surprisingly little is known about the phenomenon. Information gaps exist in almost all critical areas, including understanding its aetiology, epidemiology, and risk factors, how best to prevent and manage it, and how to optimize care delivery. The European Commission has identified multimorbidity as a key priority and is working to raise awareness, encourage innovation, optimize the use of existing resources, and coordinate the efforts of all stakeholders working in the field. Many EU countries have already prioritized multimorbidity in their national health strategies, and work is underway to improve its management. Integrated care approaches to managing patients with multimorbidity may prove to be cost-effective, but the evidence is currently limited, and these approaches may not be universally applicable. The bottom line is that we are now in the uncomfortable position of knowing we urgently need to do something about multimorbidity, but without sufficient basic information to guide us, we may not know exactly what that is.

## Figures and Tables

**Table 1:**
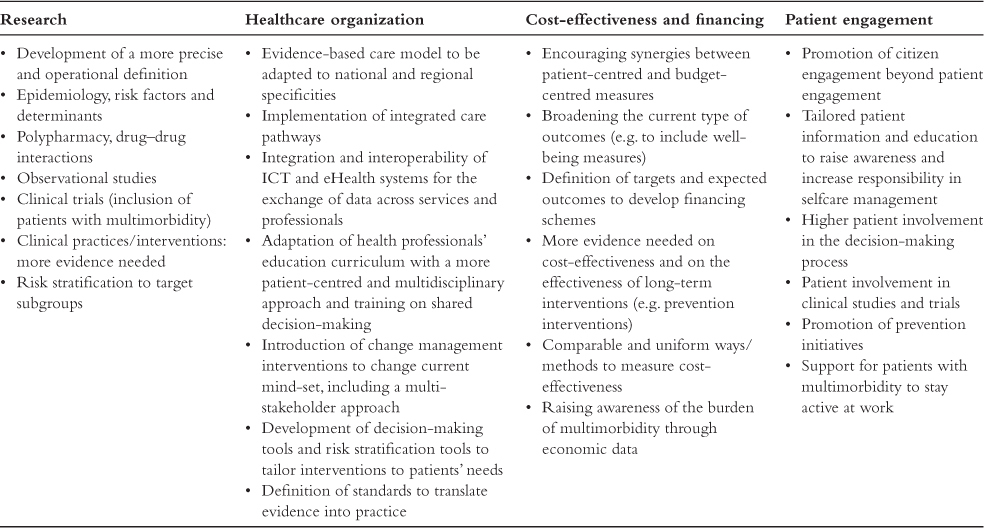
Priorities to be addressed in order to build a common European framework on multimorbidity [28].

eHealth, electronic health; ICT, information and communications technologies.
